# Mismatch repair deficient hematopoietic stem cells are preleukemic stem cells

**DOI:** 10.1371/journal.pone.0182175

**Published:** 2017-08-02

**Authors:** Yulan Qing, Stanton L. Gerson

**Affiliations:** Case Comprehensive Cancer Center, National Center for Regenerative Medicine, Case Western Reserve University, Cleveland, Ohio, United States of America; Emory University, UNITED STATES

## Abstract

Whereas transformation events in hematopoietic malignancies may occur at different developmental stages, the initial mutation originates in hematopoietic stem cells (HSCs), creating a preleukemic stem cell (PLSC). Subsequent mutations at either stem cell or progenitor cell levels transform the PLSC into lymphoma/leukemia initiating cells (LIC). Thymic lymphomas have been thought to develop from developing thymocytes. T cell progenitors are generated from HSCs in the bone marrow (BM), but maturation and proliferation of T cells as well as T-lymphomagenesis depends on both regulatory mechanisms and microenvironment within the thymus. We studied PLSC linked to thymic lymphomas. In this study, we use MSH2-/- mice as a model to investigate the existence of PLSC and the evolution of PLSC to LIC. Following BM transplantation, we found that MSH2-/- BM cells from young mice are able to fully reconstitute multiple hematopoietic lineages of lethally irradiated wild-type recipients. However, all recipients developed thymic lymphomas within three and four months post transplantation. Transplantation of different fractions of BM cells or thymocytes from young health MSH2-/- mice showed that an HSC enriched fraction always reconstituted hematopoiesis followed by lymphoma development. In addition, lymphomas did not occur in thymectomized recipients of MSH2-/- BM. These results suggest that HSCs with DNA repair defects such as MSH2-/- are PLSCs because they retain hematopoietic function, but also carry an obligate lymphomagenic potential within their T-cell progeny that is dependent on the thymic microenvironment.

## Introduction

Mismatch repair (MMR) is an essential pathway for maintaining genomic integrity mainly by removing base mismatches and small insertion/deletion loops (IDLs) introduced during replication [1]. In humans, MMR gene defects have been most closely associated with hereditary nonpolyposis colorectal cancer (HNPCC) [2,3,4]. Deficiency in MMR is associated with subsequent mutation of critical downstream genes, resulting in deregulated cell proliferation and tissue-specific tumorigenesis [5,6].

Although human HNPCC patients primarily develop cancers of the gastrointestinal tract, lymphomas and leukemias have been observed in certain kindreds [7,8]. Several human patients with germline mutations in both copies of any one of the mismatch repair genes, MLH1, MSH2, or PMS2, have presented with early-onset childhood T- or B-cell malignancies [9,10,11,12,13]. MMR deficiency has also been identified in primary and secondary hematopoietic malignancies and in leukemia and lymphoma cell lines [14,15,16]. In addition, leukemia cells from a substantial proportion of children with newly diagnosed acute lymphoblastic leukemia have low or undetectable MSH2 protein levels, despite abundant wild-type *MSH2* mRNA[17]. These reports suggest that functional MMR suppresses lymphoma/leukemia development.

Mice deficient in Msh2 most commonly develop early-onset thymic lymphomas although other tumors including small intestinal tumors occur with a lower frequency at later stage [18,19], this may be analog to the early-onset childhood T-cell malignancies in humans with biallelic MSH2 mutations [12]. Immunohistochemistry with T-cell markers, and histology, showed that these thymic lymphomas are very homogeneous, predominantly of T-cell origin, characterized by a ‘‘starry sky” appearance, enlarged nuclei, reduced cytoplasm, and numerous mitotic figures [18]. Msh2-deficient thymic lymphomas are thought to represent a single histopathologic entity and the tumor homogeneity suggests specific recurring genetic events may underlie the lymphocyte transformation and expression of a malignant phenotype [20].

Thymic lymphomas have been thought to develop from disregulated differentiation and proliferation of developing thymocytes. T cells develop in the thymus from precursors that are generated in the bone marrow (BM) and continuously seed the thymus through the blood. Maturation and proliferation of T cells depend on regulatory mechanisms in the thymus where the T-progenitors must interact with the microenvironment in the thymus to be able to differentiate [21,22]. Thymus environment is also important for lymphoma development. Earlier *in vivo* transplantation experiments have shown that whole body X-irradiation exposure or leukemia virus induced thymus-dependent pre-leukemic cells which required the thymus microenvironment for progression to full malignancy [23,24,25,26]. Thymectomy at birth or young age abolished spontaneous development in a lymphoma prone mouse stain AKR [27,28]. Thymectomy also reduced the incidence of radiation induced lymphoma in C57BL mice [29]. These data were interpreted as showing that lymphoma development is the final outcome of a series of events in which bone marrow-derived thymocyte progenitors are transformed in the thymic environment [30].

Hematopoietic stem cells (HSCs) are responsible for generation and maintenance of multiple lineages in the blood supply [31]. Recent evidence suggests that, in hematological malignancies, whereas transformation events may occur at different developmental stages, the initial mutation often originates in the HSCs, and creates a preleukemic stem cell (PLSC) [32]. PLSCs retain the ability to differentiate into the full spectrum of mature myeloid and lymphoid cells. Subsequent mutations at either stem cell or progenitor cell levels would transform the PLSC into leukemia stem cell (LSC) or leukemia initiating cells (LIC)[33,34].

MSH2-deficient cells display a mutator phenotype due to lack of MMR capacity [35], HPCs from MSH2-/- BM after serial transplantation display microsatellite instability (MSI) [36]. Though hematopoiesis in young MSH2-deficient mice appears normal, the high incidence of thymic lymphomas [18,19] in MSH2-deficient mice lead us to hypothesize that MSH2-deficient HSCs are PLSCs, and the transformation of MSH2-deficient PLSC into LSC or LIC requires the thymus microenvironment. In this study, we investigate the function of MSH2-/- HSCs, the cellular source of lymphomas, and the role of the thymic microenvironment in lymphoma development in MSH2-/- mice.

## Materials and methods

### Mice

The C57BL/6 (CD45.2) and congenic strains B6.SJL-Ptprc^a^Pep3^b^/BoyJ (BoyJ, CD45.1) mice were obtained from Jackson Laboratory. MSH2+/- mice were kindly provided by Dr. T. W. Mak. Thymectomized BoyJ mice were obtained from NCI Frederick. All the mice were housed in specific pathogen-free facility. All mouse studies were approved by the institutional animal care and use committee at Case Western Reserve University (Cleveland, OH).

### Flow cytometry and cell sorting

Flow cytometry was performed on a BD LSRI or LSRII (BD Biosciences, San Jose, CA), and data were analyzed using FlowJo software (TreeStar, Ashland, OR). Antibodies include CD45.2, CD45.1, Ly-6G (Gr-1), CD11b (Mac-1), CD45R/B220, CD4 (L3T4), CD8 (Ly2), and Ter119/Ly76, Sca1 (Ly-6A/E), c-Kit (CD117), CD34, CD16/32, IL7Ra (BD Bioscience).

BD Aria was used for cell sorting. BM cells were lineage depleted using a lineage depletion kit (Miltenyi Biotec, Auburn, CA), and labeled with phycoerythrin (PE)-conjugated lineage antibodies, fluorescein isothiocyanate (FITC)-conjugated Sca-1 and allophycocyanin (APC)-conjugated c-Kit, LSK cells were sorted.

### Transplantation assay

Single cell suspension was prepared from BM, thymus, and spleen. For BM transplantation, whole BM cells from WT or MSH2-/- mice were injected into lethally irradiated (11Gy) recipient mice through the lateral tail vein. For transplantation with thymocytes or splenocytes, isolated thymocytes or splenocytes were mixed with 2x10^5^ BM cells from BoyJ mice, and transplanted into lethally irradiated BoyJ recipients. For transplantation with lymphoma cells, isolated single cells from lymphomas were transplanted into sublethally irradiated (6Gy) BoyJ mice.

### Statistical analysis

Logrank test was used to determine the significance between survival curves. The Student’s t test was used to determine the significance in the chimerisms studies.

## Results

### Thymic lymphoma initiating cells originate from the BM hematopoietic stem/progenitor pool in MSH2-deficient mice

**Fig 1:**
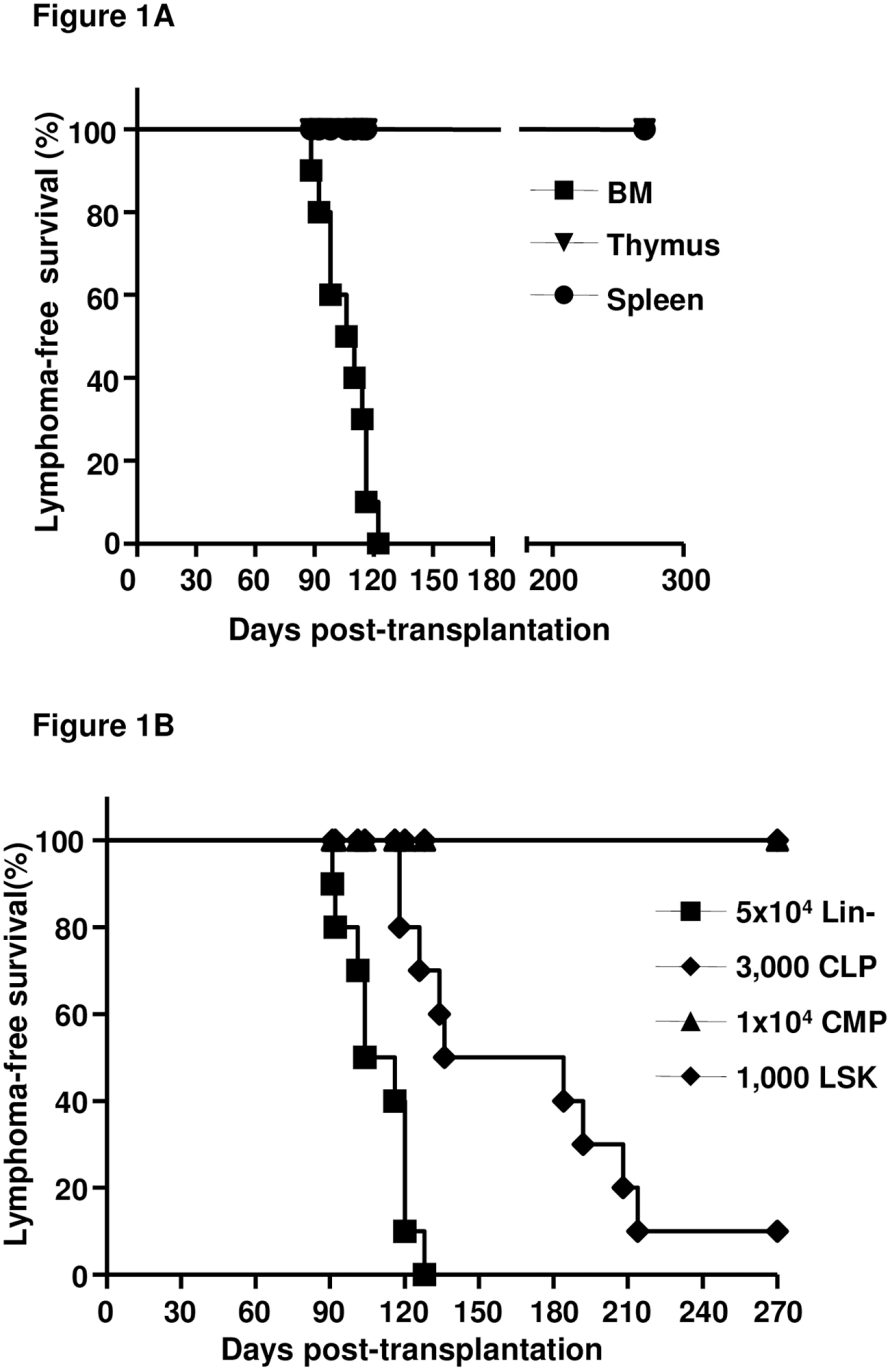
Kaplan-Meier survival curves of the recipients of transplantation with different cellular source of MSH2-/- mice. (A). 2x10^6^ cells from BM, thymus and spleen of MSH2-/- mice (n = 3) were transplanted into lethally irradiated BoyJ mice (n = 10 per group). The development of thymic lymphomas in the recipients was monitored. (B). Lineage depleted BM cells (Lin-), sorted LSK, CLP or CMP cells were transplanted along with 2x10^5^ BM cells from BoyJ mice into lethally irradiated BoyJ mice (n = 10 per group). The development of thymic lymphomas in the recipients was monitored.

To determine the cellular source of the thymic lymphomas in MSH2-deficient mice, 2x10^6^ cells from several hematopoietic tissues including BM, thymus, and spleen, of young healthy (6–8 weeks old) mice were transplanted into WT mice and thymic lymphoma development was monitored. All recipients receiving MSH2-/- BM cells developed thymic lymphomas within 3 to 4 months, while no lymphomas were observed in recipients of MSH2-/- thymocytes or splenocytes up to 9 months (Fig 1A). However, even when 1x10^7^ thymocytes or splenocytes were transplanted into WT recipients, no lymphomas or other types of hematopoietic malignancies were observed after up to 9 months (data not shown). BM transplantation with different cell doses was also performed, and recipients of all cell doses from 1x10^6^ to 1x10^5^ developed T-lymphomas, and recipients of 1x10^5^ MSH2-/- BM cells developed thymic lymphomas with slightly longer, but not significant, latency compared to the recipients of 1x10^6^ (S1 Fig). These results indicate that the cellular source of thymic lymphoma in MSH2-/- mice is in the BM.

The lymphomas observed all resulted in enlarged thymus (data not shown). The immunophenotypes of these lymphomas are similar in MSH2 -/- mice and in irradiated normal recipients transplanted with MSH2-/- BM cells. The predominant phenotype was CD4+CD8+ double positive (DP) cells (13/20 lymphomas in MSH2-/- mice vs 14/26 lymphomas of the BMT recipients). Some showed a mixed population of DP and single positive populations (5/20 MSH2-/- mice vs 9/26 BMT recipients). A minority showed a large proportion of DN thymic cells (2/20 MSH2-/- lymphomas vs 3/26 lymphomas in BMT recipients of MSH2-/- BM cells (S2 Fig). These results suggest that lymphoma development in BMT recipients is similar to lymphomagenesis in MSH2-/- mice, predominantly evolving from DP cells; and lymphomas develop from different stages of T cells in the thymus.

To further identify the cellular population leading to the development of thymic lymphomas, BM from MSH2-/- mice were fractioned based on surface markers [37], and transplanted into WT BoyJ mice (n = 10 per group). MSH2-/- mice displayed similar frequencies of HSC/progenitors (Lin-, Sca1+,c-Kit+, LSK), common lymphoid progenitor (CLP, Lin-, CD127+, Sca1^med^, c-Kit^med^) and common myeloid progenitor (CMP, Lin-, Sca1-, c-Kit+, CD34^+/low^,CD16/32^int^) compared to WT mice (S3 Fig). All the recipients of MSH2-/- Lin^-^ cells and 90% of MSH2-/- LSK cells recipients developed thymic lymphomas, while no lymphomas were observed in recipients of CLP or CMP cells up to 9 months (Fig 1B). Recipients of T cells (CD3+), B cells (B220+) or myeloid cells (Mac1+) did not develop hematopoietic malignancies of any type (data not shown). We also note that recipients of 5x10^4^ Lin^-^ cells developed T-lymphomas with similar latency to that of 2x10^6^ whole BM recipients (median latency, 110 days vs 108 days), while the latency in recipients of LSK cells was significantly longer than BM recipients (median latency, 160 days vs 108 days, P<0.0005) (Fig 1B). These results suggested that thymic lymphomas in MSH2-/- mice derived from HSC/progenitor cells, rather than lymphoid specific progenitors.

### MSH2-/- HSCs are able to reconstitute hematopoiesis in lethally irradiated recipients

**Fig 2:**
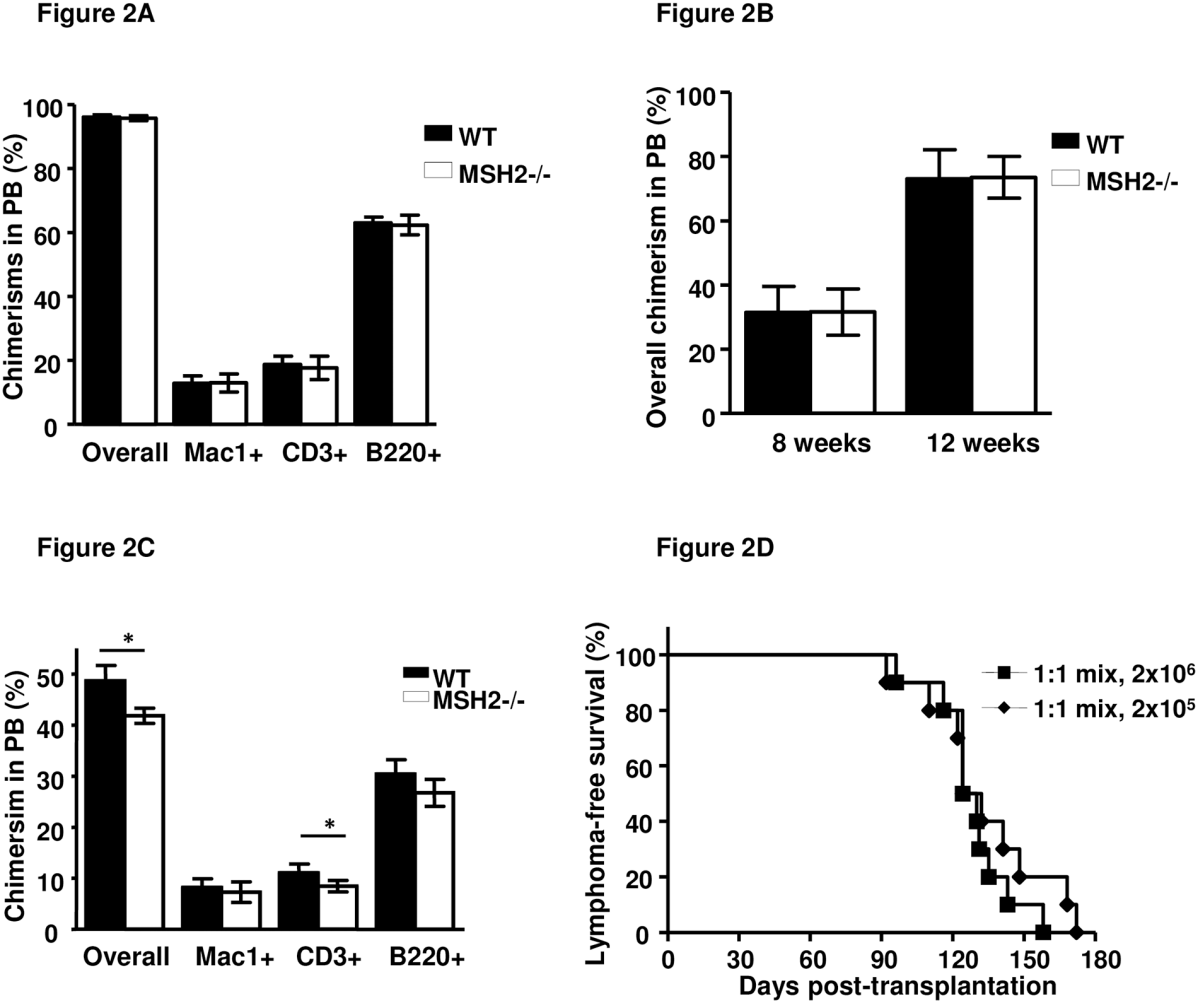
Functional assessment of HSCs from young healthy MSH2-/- mice. (A). 2x10^6^ whole BM pooled from 6–8 weeks old WT (n = 3) or MSH2-/- (n = 3) donor mice (CD45.2) into irradiated recipients (CD45.1, n = 5 per group). 8 weeks after transplantation, donor chimerism in the peripheral blood was analyzed and quantitated. Similar results were obtained in three independent experiments. (B). 1x10^3^ sorted LSK cells from 6–8 weeks old WT or MSH2-/- (n = 3 each) donor mice (CD45.2) along with 2x10^5^ BoyJ BM cells were transplanted into irradiated recipients (CD45.1, n = 10 per group). 8 and 12 weeks after transplantation, donor chimerism in the peripheral blood was analyzed and quantitated. Similar results were obtained in two independent experiments. (C). BM from 6–8 weeks old WT and MSH2-/- mice (CD45.2) was harvested and mixed with WT BM (CD45.1) at 1:1 ratio and transplanted into lethally irradiated WT mice (CD45.1, n = 10 per group). 8 weeks after transplantation, donor chimerism in the peripheral blood was analyzed and quantitated. Similar results were obtained in two independent experiments. Error bars indicate the SD, and significance was determined by a two-tailed t test. Asterisk, P<0.05. (D). Survival curve of the recipients of the experimental group described in (C).

We have previously shown that MSH2-/- HSCs are defective in repopulation in serial transplantation by CFU assays [36], which mainly assess the myelopoiesis potential of progenitors. To assess the hematopoietic reconstitution potential of MSH2-/- HSCs including both myelopoiesis and lymphopoiesis, 2x10^6^ BM cells from WT and MSH2-/- mice were transplanted into WT recipients. Similar to the results from WT BM transplantation, MSH2-/- BM contributed more than 95% overall chimerism in the peripheral blood of the recipients eight weeks post transplantation, and levels of MSH2-/- derived donor chimerism of each lineage including myeloid, T and B cells were similar to those from WT BM donor (Fig 2A). We could not determine the long-term hematopoiesis function of MSH2-/- HSCs in this setting due to the high incidence of fatal lymphoma development between 12 and 18 weeks (Fig 1). Purified LSK cells were also transplanted into WT recipients to examine their hematopoiesis activity. The contribution of MSH2-/- LSK cells to hematopoiesis was comparable to WT LSK cells at 8 and 12 weeks post transplantation (Fig 2B), and the recipients developed T-lymphomas 16–30 weeks post transplantation (Fig 1B), suggesting that the hematopoietic reconstitution function of MSH2-/- HSCs is intact, and lymphoma initiating cells evolved from the HSC/progenitor cells after hematopoietic reconstitution.

To determine if hematopoiesis and lymphomagenesis initiated by MSH2-/- BMT would be suppressed by WT BM, competitive repopulation assays were performed. When mixed with WT BM at 1:1 ratio, MSH2-/- BM contributed slightly less to the overall chimerism in the peripheral blood of the recipients 8 weeks post transplantation compared to WT BM (41.8+/-1.5% vs 48.6+/-3.0%) (Fig 2C), with similar contributions to myeloid (7.3+/-2.0% vs 8.2+/-1.7%) and B cell (26.7+/-2.6% vs 30.4+/-2.8%), but slightly less contribution to T cell (8.4+/-1.1% vs11.1+/-1.7%) chimerism (Fig 2C). All recipients developed MSH2-/- BM derived lymphomas (Fig 2D). Mice with signs of lymphomas including labored breathing, loss of body weight and mobility, were examined for donor chimerism in the peripheral blood and thymus. While about 50% of the leukocytes in the peripheral blood derived from MSH2-/- cells, more than 95% of the cells in the lymphomas are MSH2-/-, and as noted, most of the lymphomas are double positive for CD4 and CD8 (S4 Fig). Interestingly, lymphoma latencies from mixtures of 2x10^5^ and 2x10^6^ total cells are similar (median latency, 127days vs 128 Days, P = 0.39) (Fig 2D). These results indicate that MSH2-/- HSCs were not defective in full hematopoietic repopulation—and thus of HSC function—compared to WT HSCs. However, whereas MSH2-/- cells were responsible for initiating T-lymphomas.

### MSH2-/- thymic lymphomas are transplantable

To determine whether the T-lymphomas are transplantable, MSH2-/- mice bearing lymphomas were used as donors for transplantation. Lymphoma cells were transplanted into sublethally irradiated WT mice at different cell doses, as few as 40 cells was able to initiate T-cell leukemia within 5 weeks (Table 1).

**Table 1:** Lymphoma/T-leukemia development in secondary recipients.

Cell doses	T-cell lymphoma/leukemia incidence	Latency (days)
**Experiment #1**		
**4x10**^**5**^	**5/5**	**14–18**
**4x10**^**4**^	**5/5**	**14–20**
**4x10**^**3**^	**5/5**	**20–24**
**400**	**5/5**	**22–25**
**40**	**2/5**	**35–38**
**Experiment #2**		
**4x10**^**4**^	**5/5**	**15–18**
**4x10**^**3**^	**5/5**	**18–26**
**400**	**5/5**	**24–28**
**40**	**0/5**	
**Experiment #3**		
**4x10**^**4**^	**5/5**	**14–16**
**4x10**^**3**^	**5/5**	**14–20**
**400**	**5/5**	**20–24**
**40**	**1/5**	**34**

Indicated numbers of lymphoma cells were transplanted into sublethally irradiated WT recipients (n = 5 per group). Lymphoma development in the recipients was monitored. Similar results were obtained in three independent experiments.

**Fig 3:**
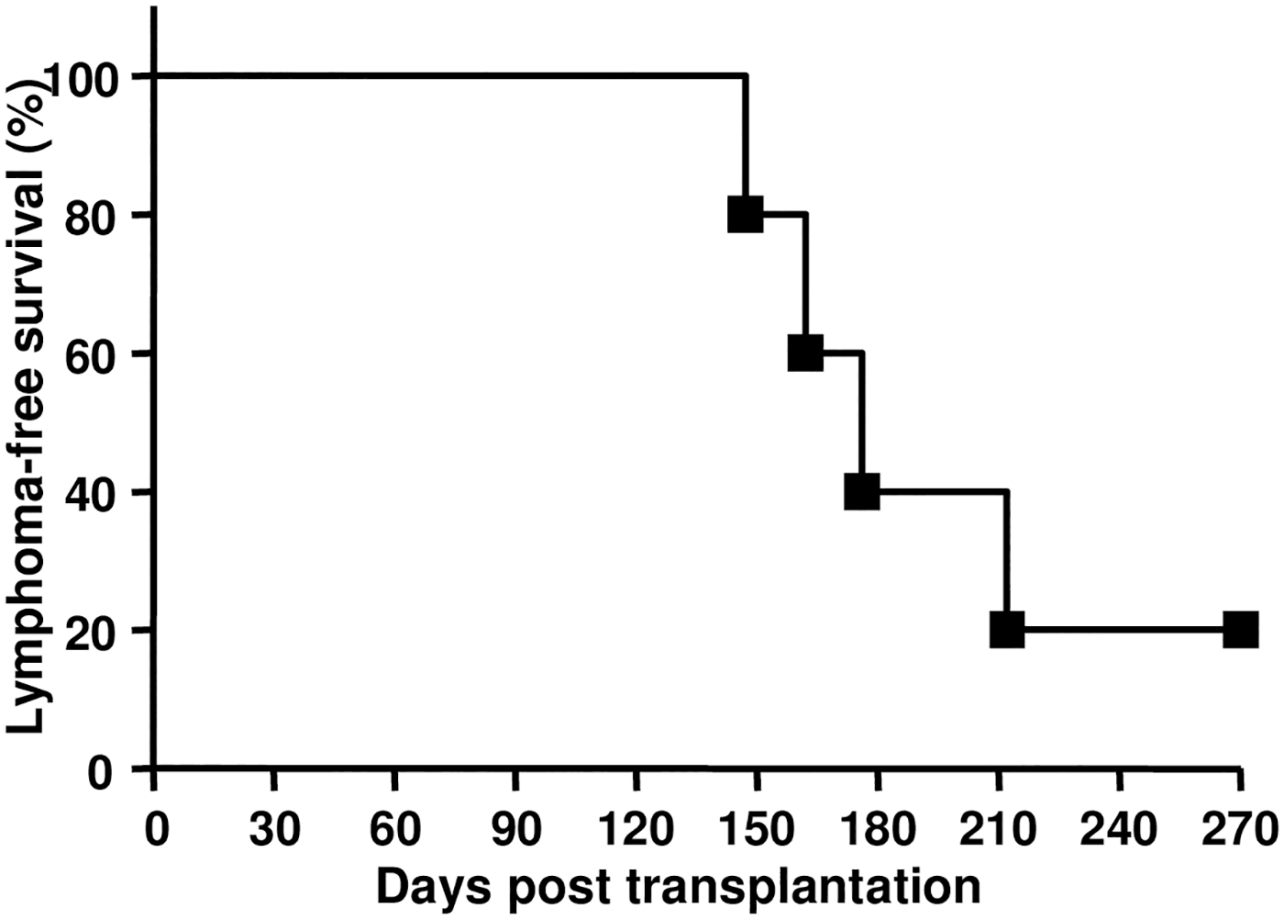
Functional assessment of MSH2-/- HSCs from lymphoma bearing mice. 1x10^3^ sorted LSK cells from MSH2-/- mouse with lymphoma symptoms along with 2x10^5^ BoyJ BM cells were transplanted into irradiated recipients (n = 10 per group). The development of thymic lymphomas in the recipients was monitored.

To determine if the HSCs in the mice with lymphomas have increased lymphomagenetic potential, 1x10^3^ sorted LSK cells were transplanted into lethally irradiated WT mice. LSK cells contributed to mutlilineage hematopoiesis (data not shown), and 80% of the recipients developed T-lymphomas with a mean latency of 176 days post-transplantation (Fig 3). A similar T-lymphoma phenotype and latency was observed after transplanting HSC-LSK cells isolated from young healthy and from the lymphoma-bearing MSH2-/- mice (Figs 1B and 3) suggested that HSC/progenitors of MSH2-/- mice retained hematopoietic function and were not altered by the presence of thymic lymphomas.

### Thymus is required for the development of thymic lymphomas in MSH2-/- mice

**Fig 4:**
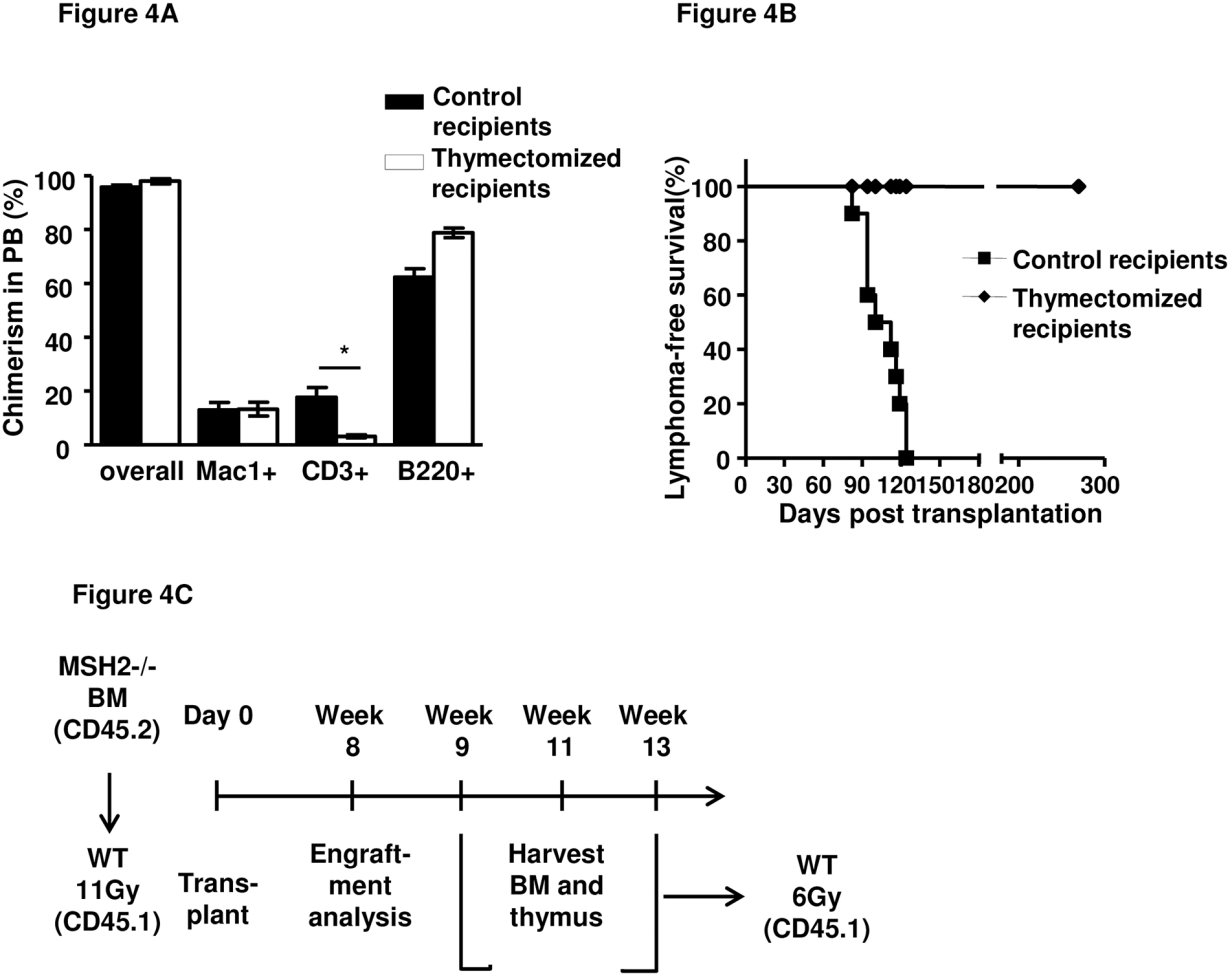
Requirement of thymus for the development of MSH2-/- BM derived thymic lymphomas. (A). 2x10^6^ BM cells from 6–8 weeks old MSH2-/- mice (n = 3) were transplanted into lethally irradiated WT control or thymectomized mice (n = 10 per group). 8 weeks after transplantation, donor chimerism in the peripheral blood was analyzed and quantitated. Similar results were obtained in two independent experiments. Error bars indicate the SD, and significance was determined by a two-tailed t test. (B). Survival curve of the recipients of the experimental group described in (A). (C). Scheme of the transplantation to determine the initial appearance of lymphoma initiating cells.

All the tumors were thymus-centric, suggesting a strong dependence on signals received from the thymic environment and that lymphoma initiating cells may evolve in the thymus. To determine whether the thymus was necessary for T-lymphoma development, MSH2-/- BM cells were transplanted into thymectomized irradiated WT recipients. Reduced T-cell reconstitution was observed in the recipients due to the lack of host thymus (Fig 4A). While MSH2-/- lymphomas developed 3–4 months post-transplantation in thymus-intact WT recipients, no T-lymphomas was observed in thymectomized recipients of MSH2-/- BM cells observed for up to 9 months (Fig 4B).

We also tested the requirement of the thymus for the expansion of lymphoma cells. T-cells were taken from the lymphomas, and transplanted into control and thymectomized mice. Each of the control and thymectomized recipients rapidly developed lymphoma/leukemia with a median latency of 15 days (S5 Fig). These results indicate that lymphoma cells do not need the thymus to propagate or expand, but that the thymus was required for the evolution of lymphoma initiating cells (LICs).

Next, we tested whether the evolution of LICs from normal HSC occurred in the thymus. 2x10^6^ MSH2-/- BM cells were transplanted into irradiated WT recipients, 8 weeks post transplantation and hematopoietic reconstitution by MSH2-/- BM was confirmed (data not shown). Thymus and BM from the primary recipients were harvested at 9, 11, and 13 weeks post transplantation, and transplanted into sublethally irradiated WT secondary recipients (Fig 4C). While secondary recipients of BM cells at all three time points developed T-lymphomas 4–6 months post transplantation, all the recipients of thymocytes harvested from primary recipients at 9 weeks post transplantation developed T-cell leukemia within 8 weeks, whereas recipients of thymocytes harvested from primary recipients at 11 and 13 weeks post transplantation developed T-cell leukemia within 4 weeks (Table 2). The significantly shorter latency of lymphoma/leukemogenesis observed after thymocyte transplantation compared to the BM recipients reaffirmed that the thymus is the site of, and is required for, the evolution of LICs.

**Table 2:** Thymocytes and BM from primary recipients of MSH2-/- BM induced lymphoma/T-leukemia in secondary recipients.

Donor Cell source	T-cell lymphoma/leukemia incidence	Latency (days)	Median Latency (days)
**BM from primary recipients at weeks post transplantation**			
**9 weeks**	**5/5**	**128–186**	**154**
**11 weeks**	**5/5**	**122–174**	**150**
**13 weeks**	**5/5**	**104–188**	**152**
**Thymocytes from primary recipients at weeks post transplantation**			
**9 weeks**	**5/5**	**33–57**	**48**
**11 weeks**	**5/5**	**12–28**	**26**
**13 weeks**	**5/5**	**21–27**	**23**

2x10^6^ MSH2-/- BM cells were transplanted into WT irradiated recipients. Thymus and BM from the primary recipients were harvested at 9, 11, and 13 weeks post transplantation (pooled from three mice each group), and 2x10^6^ BM cells or thymocytes were transplanted into sublethally irradiated WT secondary recipients (n = 5 per group). Lymphoma/leukemia development in the secondary recipients was monitored. Similar results were obtained in two independent experiments.

**Fig 5:**
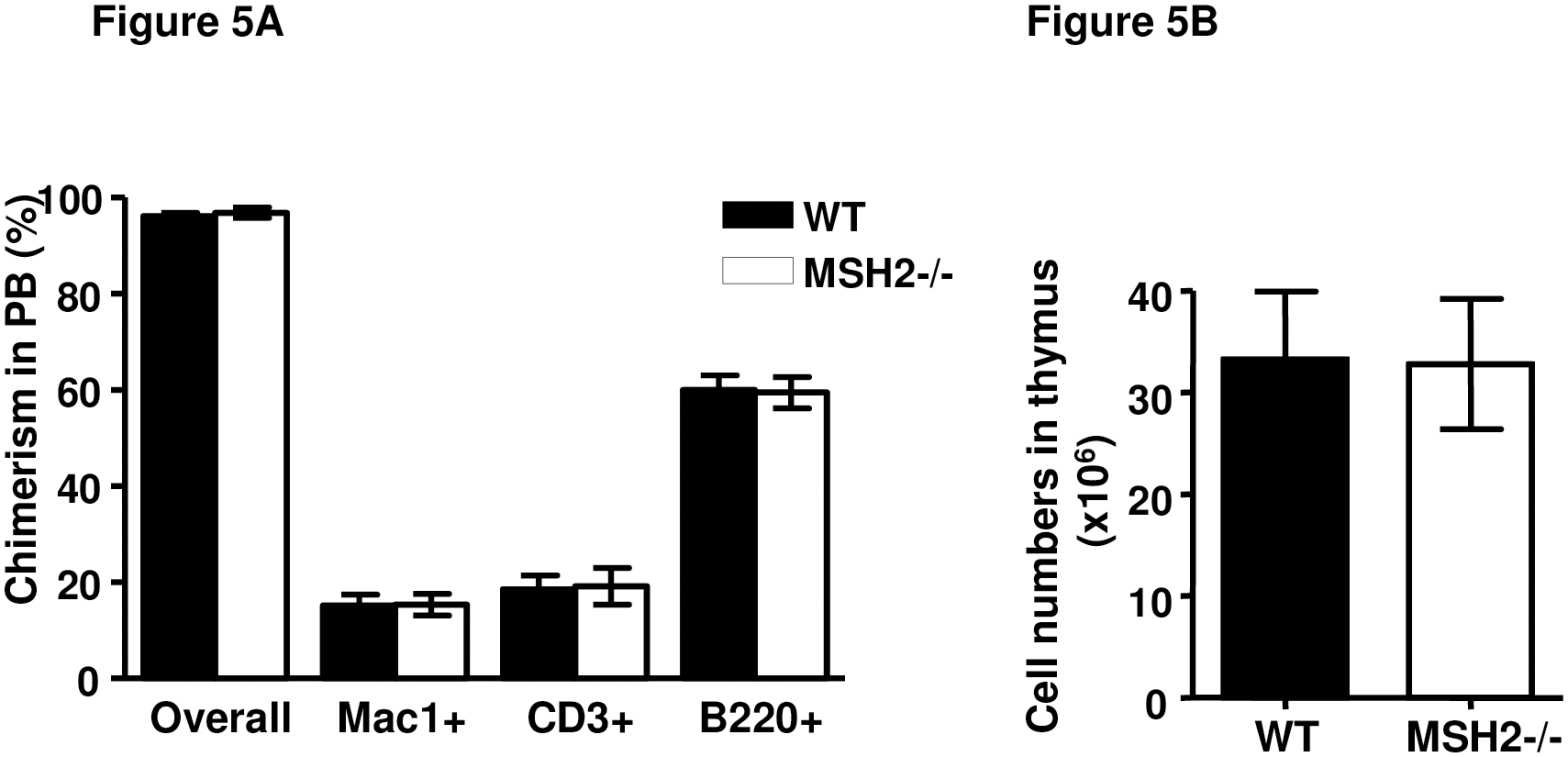
Normal thymus microenviroment of MSH2-/- mice. (A).2x10^6^ BM cells from BoyJ mice (n = 3) were transplanted into 6–8 weeks old WT or MSH2-/- mice (n = 10 per group). 8 weeks after transplantation, donor chimerism in the peripheral blood was analyzed and quantitated. (B) Total cell numbers within thymus of the recipients at 8 weeks post transplantation. Error bars indicate the SD, and significance was determined by a two-tailed t test.

To further determine the impact of the microenvironment in MSH2-/- mice on lymphomagenesis, 2x10^6^ WT BM cells were transplanted into lethally irradiated MSH2-/- mice, resulting in WT BM reconstitution of hematopoiesis with more than 95% overall chimerism in the peripheral blood of recipients, and normal lineage (Fig 5A). No recipients developed T-lymphomas or other hematopoietic malignancies observed for up to 9 months (data not shown). WT BM derived-T cell development within the thymus of MSH2-/- mice was also examined at 8 weeks post transplantation. Within the thymus, the total cell numbers (Fig 5B) and composition of T-cell subsets are comparable to WT controls (S6 Fig). These results indicated that the MSH2-/- BM microenvironment is not impaired in supporting hematopoiesis and that the MSH2-/- thymus microenvironment does not promote lymphomagenesis from WT progenitors.

## Discussion

The primary purpose of this study was to investigate the process of HSC-derived thymic lymphomagenesis in MSH2-/- mice. We set out to determine the cellular source of the LICs and the sites at which LICs develop into thymic lymphomas. Our results provide evidence that MSH2-/- HSCs are PLSCs, and while they retain full hematopoietic potential, their progeny gain lymphomagenetic potential in the thymic microenvironment and become LICs and initiate lymphomagenesis. Thus, the HSC is an obligate precursor carrier of the LIC in MSH2-/- mice.

Though MSH2-/- mice display somatic hyper mutation defects [38], it appears that MSH2 is not involved in T cell development. When MSH2-/- BM cells were transplanted into WT recipients, the hematopoietic reconstitution was similar with those receiving WT BM cells (Fig 2A). These results suggested that MSH2 is not critical for T-cell development or hematopoietic reconstitution. When MSH2-/- BM cells were mixed with WT BM at 1:1 ratio, MSH2-/- showed modest defects in competitive repopulation activity in the primary recipients (Fig 2C). These results indicate that the hematopoiesis function of MSH2-/- HSCs was largely intact.

MSH2-/- mice develop spontaneous thymic lymphomas starting at 3 months of age, most mice die from the disease within 5–7 months old ([18,19] and our unpublished results). Using BM transplantation, we found that BM cells from young healthy MSH2-/- mice were able to initiate thymic lymphomas in the recipients within 3–4 months post transplantation; whereas thymocytes and splenocytes were not able to initiate lymphomas (Fig 1A). These results indicate that the cellular source for lymphomagenesis originates from the BM, rather than the more specific lymphoid organs. Only the fractions enriched with HSC/progenitors (Lin- and LSK cells) were able to initiate lymphomagenesis, whereas the lineage positive populations or committed progenitors were not able to do so (Fig 1B and data not shown). In fact, the lineage positive cells and CLP/CMP were not able to maintain engraftment in the recipients [39,40] (and data not shown). These results suggest that HSC/progenitors in MSH2-/- mice are the cellular sources for lymphomagenesis. It is likely that sustained engraftment and hematopoiesis are required for the progenitor cells to initiate lymphomagenesis. Importantly, the limiting dilution BM transplantation studies showed that all recipients of even 1x10^5^ BM from MSH2-/- mice developed thymic lymphomas (Fig 2C), and the frequency of HSCs in mouse BM is about 0.01% of total nucleated cells[37,41], suggesting that perhaps every HSC in MSH2-/- mice has the lymphomagenetic potential. Of note, LSK cells from lymphoma bearing mice does not cause early-onset lymphoma in the recipients (Fig 3), and are able to contribute to multilineage hematopoietic reconstitution in the recipients (data not shown), suggesting that LSK cells are not the direct source for LIC.

These results allow us to propose that HSCs in the BM of MSH2-/- mice are PLSCs, retaining full hematopoietic potential, but are an obligate carrier of full lymphomagenic potential. In this regard, the PLSCs in MSH2-/- mice have similar characteristics to LSCs in human acute myelogenous leukemia (AML). For instance, the LSCs in human AML share the immunophenotype of normal HSCs, possesses proliferation and differentiation potential[42]. An example is AML derived from AML1/ETO [43]. By surveying *AML1/ETO* mRNA expression in rigorously purified HSC, progenitors, and mature hematopoietic cells of various lineages, Miyamoto et al. demonstrated that acquisition of AML1/ETO occurs in HSC/progenitors, and that these progenitors contribute to B lymphopoiesis as well as myelopoiesis throughout the clinical course [44]. Interestingly, AML1/ETO is necessary, but not sufficient for transformation or leukemia development, and a fraction of the AML1/ETO-expressing stem cells undergo additional oncogenic event(s) that ultimately leads to transformation into AML[44,45]. Future studies of lymphomagenesis with MSH2-/- mouse model will provide important insight onto the process of human AML LSCs evolution from normal HSCs.

We also show that the thymus microenvironment is required for the MSH2-/- BM derived lymphomagenesis. While all the recipients of MSH2-/- BM cells developed lymphomas, the thymectomized recipients did not develop lymphomas or leukemias (Fig 4B). And when thymocytes and BM from the primary recipients of MSH2-/- BM transplantation were transplanted into secondary recipients, recipients of thymocytes developed lymphomas with a much shorter latency compared to BM recipients (Table 2). These results indicate that the evolution of PLSC to lymphoma requires T cell progenitors to migrate to the thymus, and thymocyte differentiation within the thymic microenvironment. On the other hand, intrinsic function of the thymic microenvironment in MSH2-/- mice appears normal, since no malignancies developed when WT BM were transplanted into MSH2-/- mice, and the development of T cells in the thymus appear normal (Fig 5 and S6 Fig).

Our observations do not appear isolated to MSH2. MLH1 is another key component in the MMR pathway. Mice deficient in MLH1 develop lymphomas at a lower frequency compared to MSH2-/- mice [46], while mice with thymocyte-specific deletion of MLH1 develop lymphomas at a remarkably reduced frequency compared to MLH1-/- mice (6% vs 26%), and most of the lymphomas are double positive [46]. These results indicate that the MMR pathway prevents genetic events that lead to cellular transformation at an early T cell development stage or an even earlier stage of hematopoiesis [47].

Loss of MMR allows cells to accumulate mutations over time. MSH2-/- hematopoietic progenitors displayed increased MSI in the BM from secondary recipients [36],MSI and mutational spectrum of the thymic lymphomas in MSH2-/- mice have been demonstrated [18,48,49,50,51]. However, a single targeted mutational lymphomagenic gene has not been identified and rather appears pleotropic. Insertion/deletion mutations of TGFbeta receptor II have been observed in lymphomas from MSH2-/-mice, the mutations correlate with loss of TGFbeta receptor II expression [52], but TGFbeta receptor II deficient thymocytes develop normally, without lymphoma incidence [53]. Expression levels of three human lymphoma-related genes LMO2, SCL and HOX11, have been examined in MSH2-/- mouse lymphomas. Expression of LMO2, SCL, and HOX11 is detected in 100%, 40%, and 0% of the cases, respectively [54]. However, whether LMO2 and SCL are direct targets of the mutator phenotype is not known. In addition, overexpression of LMO2 or SCL alone in developing thymocytes in the mouse results in the development of T-ALL with long latency, LMO2 and SCL cooperate to accelerate lymphomagenesis [55].

Lymphomagenesis is thought to require disruption of multiple pathways [56], MSH2-/- lymphomas might be a good model to identify lymphomagenic genes. In patients with Lynch syndrome and HNPCC, loss of MMR leads to many second hits, and accumulation of specific genetic alterations in MSI-positive colorectal cancers is markedly heterogeneous [57,58,59]. It is likely that MSH2-/- HSCs in mice like MMR-deficient colorectal cancer stem cells do not contain driver transformational mutations. Rather, during the expansion and differentiation process, their progeny acquire such driver mutations, with clonal expansion result in lympomagenesis. However, it is instructive to appreciate that in MSH2-/- mice, these data indicate that the HSC are, for the most part, obligate PLSC.

## Supporting information

S1 Fig Kaplan-Meier survival curves of the recipients of transplantation with different numbers of MSH2-/- BM cells. (PDF)

S2 Fig Lymphoma characterization in the recipients of MSH2-/- BMT and MSH2-/- mice. (PDF)

S3 Fig Characterization of hematopoietic progenitor pools in MSH2-/- mice. (PDF)

S4 Fig Example of immunophenotypes of thymic lymphomas in the group described in Fig 2C. (PDF)

S5 Fig Lymphoma development in the secondary thymectomized recipients. (PDF)

S6 Fig T cell composition in the thymus of MSH2-/- mice post transplantation. (PDF)
