# Supplementary material for: Mismatch repair deficient hematopoietic stem cells are preleukemic stem cells
Source: PLoS One. 2017 Aug 2;12(8):e0182175. doi: 10.1371/journal.pone.0182175 (PMC5540588; doi:10.1371/journal.pone.0182175)
Supplement: S1 Fig — (PDF) [file pone.0182175.s001.pdf]

S1 Fig

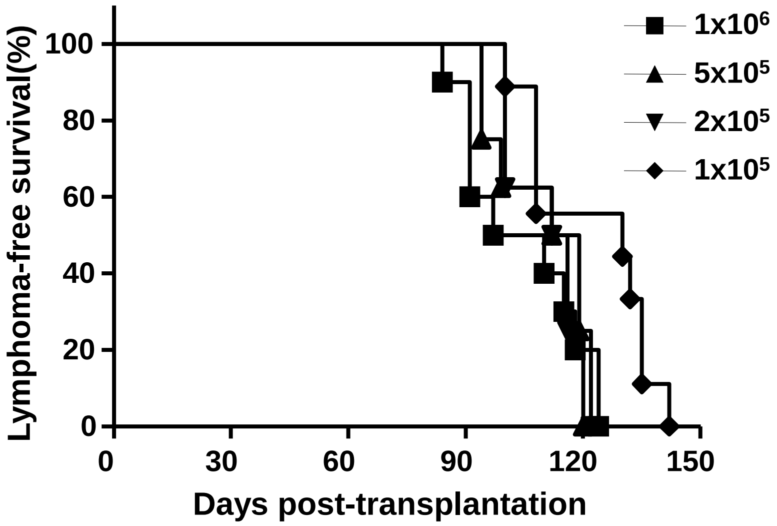

S1 Fig. Kaplan-Meier survival curves of the recipients of transplantation with different numbers of MSH2-/- BM cells. Indicated numbers of BM cells from 6-8 weeks old MSH2-/- mice (n=3) were transplanted into lethally irradiated BoyJ mice (n=10 per group). The development of thymic lymphomas in the recipients was monitored.
