# Supplementary material for: Mismatch repair deficient hematopoietic stem cells are preleukemic stem cells
Source: PLoS One. 2017 Aug 2;12(8):e0182175. doi: 10.1371/journal.pone.0182175 (PMC5540588; doi:10.1371/journal.pone.0182175)
Supplement: S2 Fig — (PDF) [file pone.0182175.s002.pdf]

S2 Fig

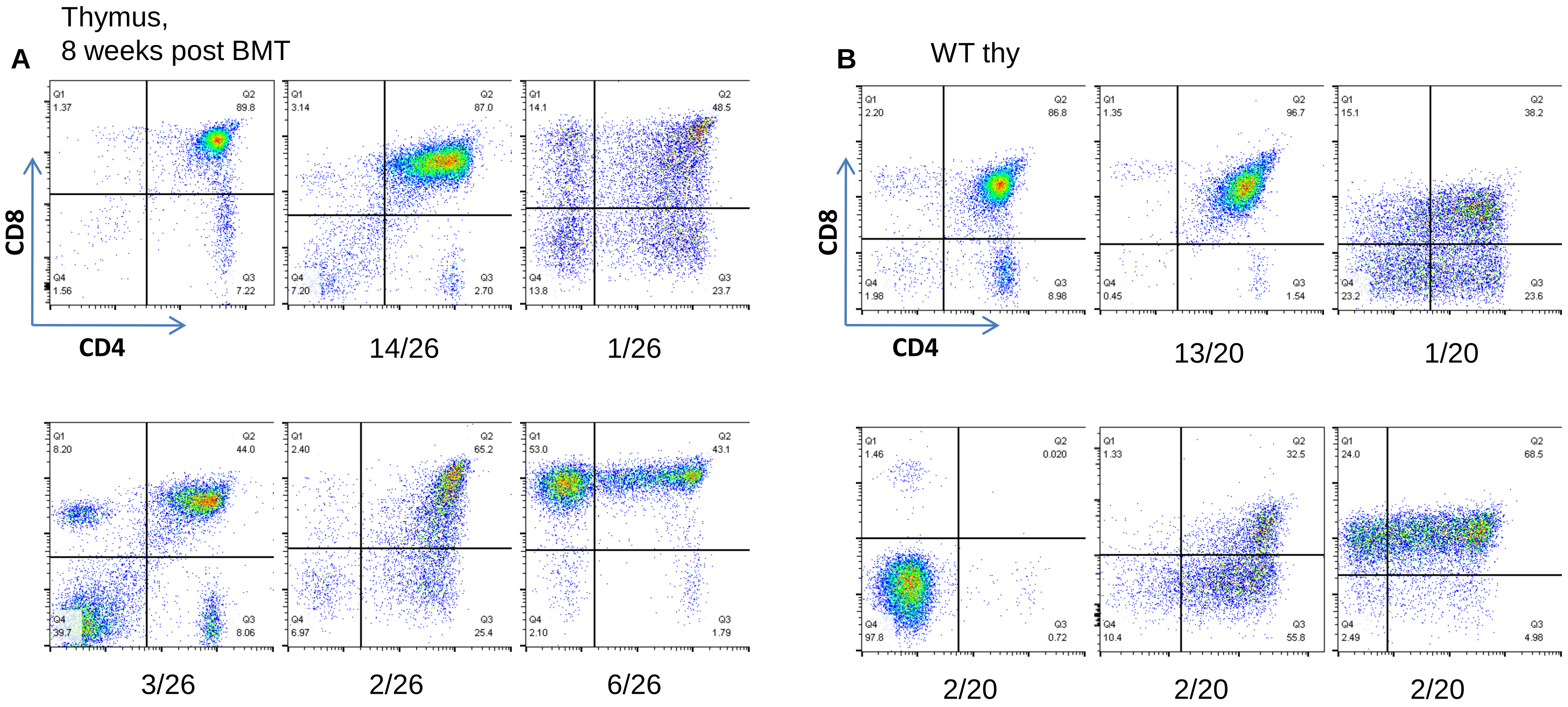

S2 Fig. Lymphoma Characterization in the recipients of MSH2-/- BMT (A) and MSH2-/- Mice (B). A total of 26 thymic lymphomas in the recipients of MSH2-/- BMT (A) and 20 lymphomas in MSH2-/- mice (B) were analyzed by flow cytometry using CD4 and CD8 antibodies. The numbers under each graph indicate the proportion of lymphomas showing similar CD4/CD8 expression profiles. The upper left graph in each panel is representative of a normal thymus from MSH2-/- BMT (A) or WT mouse (B). Note: the gating may not be based on a singular standard, since this is a long-turn experiments, the samples were analyzed at different times.
