# Supplementary material for: Mismatch repair deficient hematopoietic stem cells are preleukemic stem cells
Source: PLoS One. 2017 Aug 2;12(8):e0182175. doi: 10.1371/journal.pone.0182175 (PMC5540588; doi:10.1371/journal.pone.0182175)
Supplement: S3 Fig — (PDF) [file pone.0182175.s003.pdf]

S3 Fig

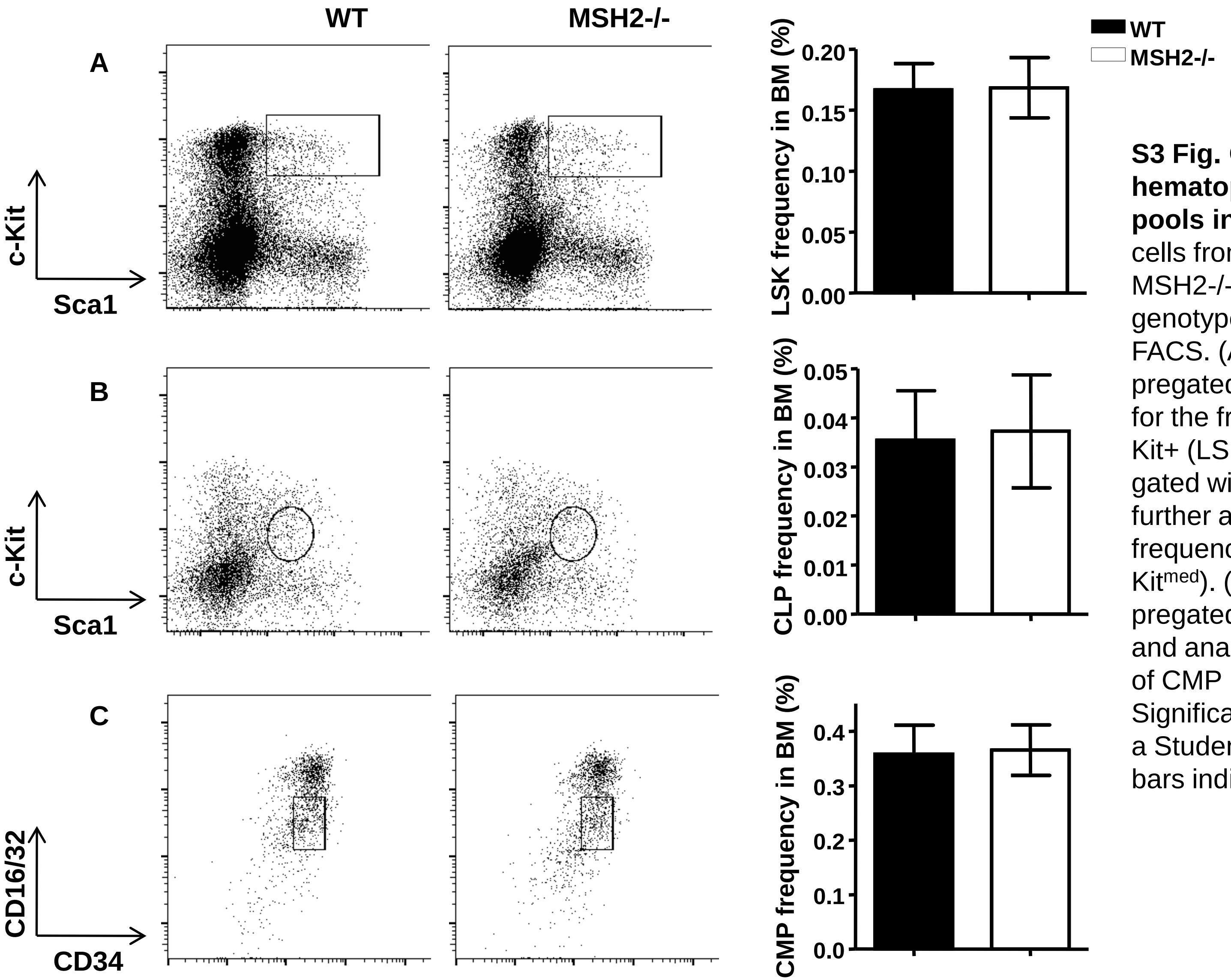

S3 Fig. Characterization of hematopoietic progenitor pools in MSH2-/- mice. BM cells from age-matched WT and MSH2-/- mice (n=6 each genotype) were analyzed by FACS. (A).BM cells were pregated for Lin-, and analyzed for the frequencies of Sca1+, c-Kit+ (LSK). (B). BM cells were gated with Lin-, CD127+, and further analyzed for the frequency of CLP (Sca1^{med}, c-Kit^{med}). (C). BM cells were pregated for Lin-, Sca1-,c-Kit+, and analyzed for the frequencies of CMP (CD34$^{+/low}$CD16/32^{int}). Significance was determined by a Student's 2-tailed t test. Error bars indicate the SD.
