# Supplementary material for: Mismatch repair deficient hematopoietic stem cells are preleukemic stem cells
Source: PLoS One. 2017 Aug 2;12(8):e0182175. doi: 10.1371/journal.pone.0182175 (PMC5540588; doi:10.1371/journal.pone.0182175)
Supplement: S4 Fig — (PDF) [file pone.0182175.s004.pdf]

S4 Fig

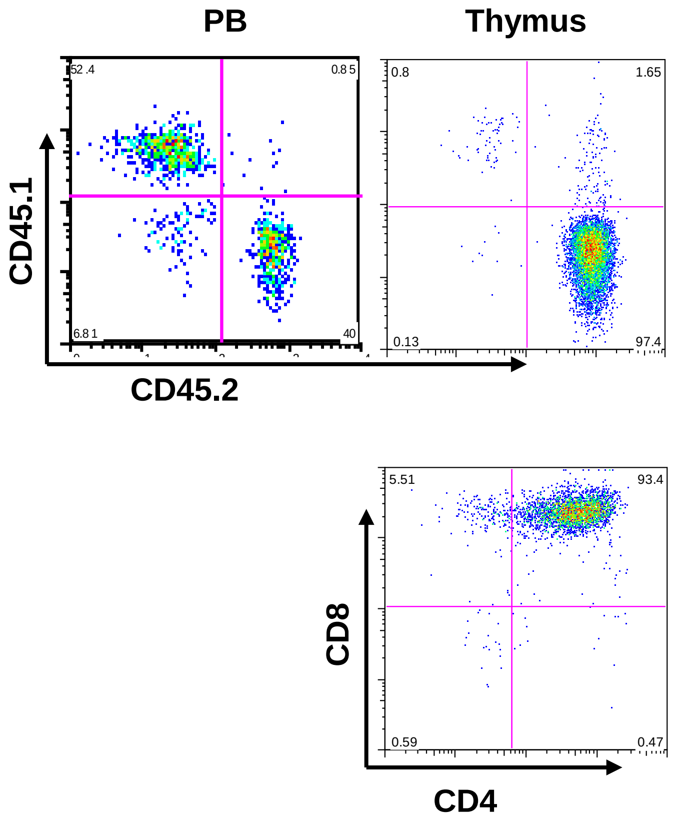

S4 Fig. Example of immunophenotypes of thymic lymphomas in the group described in Figure 2C. BM from MSH2-/- mice were mixed with WT BM cells at 1:1 ratio and transplanted into lethally irradiated BoyJ mice, lymphoma development in the recipients was monitored as described in Figs 2C and D. When the recipient mouse displayed lymphoma symptoms, peripheral blood was collected, the mouse was sacrificed and thymic lymphoma was dissected, single cell suspension was prepared, and analyzed by flow cytometry.
