# Supplementary material for: Mismatch repair deficient hematopoietic stem cells are preleukemic stem cells
Source: PLoS One. 2017 Aug 2;12(8):e0182175. doi: 10.1371/journal.pone.0182175 (PMC5540588; doi:10.1371/journal.pone.0182175)
Supplement: S5 Fig — (PDF) [file pone.0182175.s005.pdf]

S5 Fig

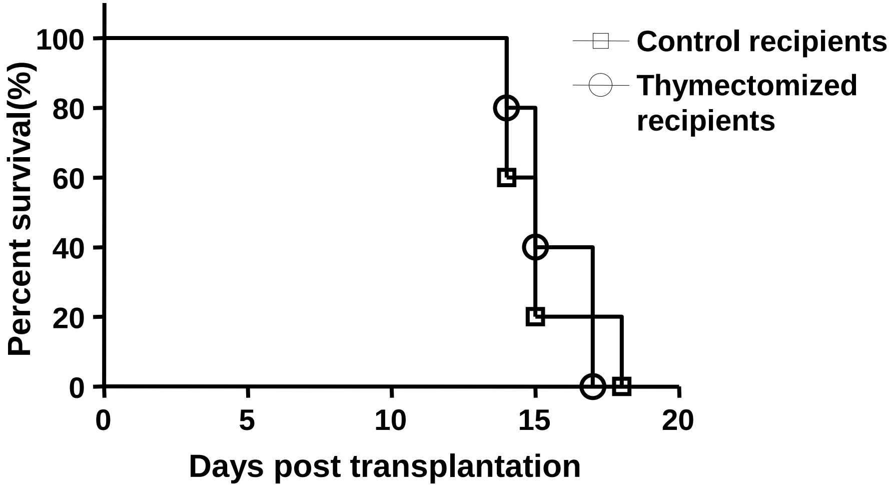

S5 Fig. Lymphoma development in the secondary thymectomized recipients. $4x10^4$ lymphoma cells were transplanted into sublethally irradiated WT control and thymectomized recipients (n=5 per group). Lymphoma development in the recipients was monitored. Similar results were obtained in three independent experiments.
