# Supplementary material for: Mismatch repair deficient hematopoietic stem cells are preleukemic stem cells
Source: PLoS One. 2017 Aug 2;12(8):e0182175. doi: 10.1371/journal.pone.0182175 (PMC5540588; doi:10.1371/journal.pone.0182175)
Supplement: S6 Fig — (PDF) [file pone.0182175.s006.pdf]

S6 Fig

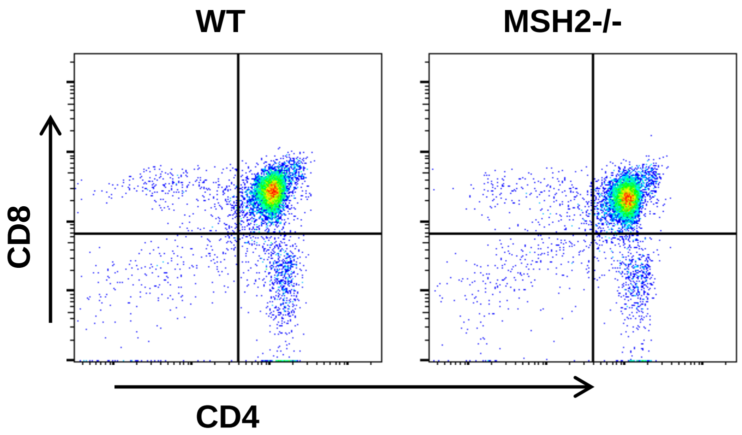

S6 Fig. T cell composition in the thymus of MSH2-/- mice post transplantation. BM cells from BoyJ mice were transplanted into WT or MSH2-/- mice as described in Figure 5. 8 weeks after transplantation, recipient mice (n=5 per group) were sacrificed, thymus tissues were dissected, single cell suspension was prepared, and the expression of CD4 and CD8 on the thymocytes was analyzed by flow cytometry.
